# Small-molecule BCL6 inhibitor protects chronic cardiac transplant rejection and inhibits T follicular helper cell expansion and humoral response

**DOI:** 10.3389/fphar.2023.1140703

**Published:** 2023-03-17

**Authors:** Yuxuan Xia, Sheng Jin, Yuming Wu

**Affiliations:** ^1^ Department of Physiology, Hebei Medical University, Shijiazhuang, Hebei, China; ^2^ Hebei Collaborative Innovation Center for Cardio-cerebrovascular Disease, Shijiazhuang, Hebei, China

**Keywords:** BCL6, FX1, cardiac transplant rejection, T follicular helper cells, humoral response

## Abstract

**Background:** B cell lymphoma 6 (BCL6) is an important transcription factor of T follicular helper (Tfh) cells, which regulate the humoral response by supporting the maturation of germinal center B cells and plasma cells. The aim of this study is to investigate the expansion of T follicular helper cells and the effect of the BCL6 inhibitor FX1 in acute and chronic cardiac transplant rejection models.

**Methods:** A mouse model of acute and chronic cardiac transplant rejection was established. Splenocytes were collected at different time points after transplantation for CXCR5^+^PD-1^+^ and CXCR5^+^BCL6^+^ Tfh cells detection by flow cytometry (FCM). Next, we treated the cardiac transplant with BCL6 inhibitor FX1 and the survival of grafts was recorded. The hematoxylin and eosin, Elastica van Gieson, and Masson staining of cardiac grafts was performed for the pathological analysis. Furthermore, the proportion and number of CD4^+^ T cells, effector CD4^+^ T cells (CD44^+^CD62L^−^), proliferating CD4^+^ T cells (Ki67^+^), and Tfh cells in the spleen were detected by FCM. The cells related to humoral response (plasma cells, germinal center B cells, IgG1^+^ B cells) and donor-specific antibody were also detected.

**Results:** We found that the Tfh cells were significantly increased in the recipient mice on day 14 post transplantation. During the acute cardiac transplant rejection, even the BCL6 inhibitor FX1 did not prolong the survival or attenuate the immune response of cardiac graft, the expansion of Tfh cell expansion inhibit. During the chronic cardiac transplant rejection, FX1 prolonged survival of cardiac graft, and prevented occlusion and fibrosis of vascular in cardiac grafts. FX1 also decreased the proportion and number of splenic CD4^+^ T cells, effector CD4^+^ T cells, proliferating CD4^+^ T cells, and Tfh cells in mice with chronic rejection. Moreover, FX1 also inhibited the proportion and number of splenic plasma cells, germinal center B cells, IgG1^+^ B cells, and the donor-specific antibody in recipient mice.

**Conclusion:** We found BCL6 inhibitor FX1 protects chronic cardiac transplant rejection and inhibits the expansion of Tfh cells and the humoral response, which suggest that BCL6 is a potential therapeutic target of the treatment for chronic cardiac transplant rejection.

## 1 Introduction

The Antibody-mediated rejection (AMR) limited long-term survival of graft in heart transplant patients (Miyairi et al., 2021; Kervella et al., 2022). However, the mechanism of AMR has not been fully elucidated and methods to prevent the AMR after heart transplantation need improve. Currently, there is not a standard treatment for AMR, since multiple immune cells are participating the course, such as T cells, B cells and macrophages (Lee, 2017). Furthermore, identifying patients who need the treatment for AMR is difficult, especially in patients suffering from asymptomatic AMR (Montgomery et al., 2018). It has been reported that AMR occurs in one-half of cardiac transplant recipients (Carbone, 2018). Therefore, it is critical to elucidate the mechanism of AMR in cardiac transplant rejection. T follicular helper (Tfh) cell is a subset of CD4^+^ T cells and promote the AMR after transplantation (Nurieva et al., 2008; Laguna-Goya et al., 2019). Tfh cells stimulate the proliferation of B cells and the transformation of immunoglobulins by cytokines production (Vinuesa et al., 2016; Niogret et al., 2021). They also promote B cell to differentiate into plasma and memory B cells by stimulating germinal centers (GCs) in humoral immunity (Vinuesa et al., 2016; Kanellopoulos and Ojcius, 2019). In this way, Tfh cells serve as the bridge between cellular and humoral immunity. Tfh cells are likely to be associated with the occurrence of AMR, and they are potential targets for AMR intervention (Li et al., 2018; Louis et al., 2021). An *in-vitro* study revealed that the Tfh cells promoted the humoral immunity in recipient and cultivated the AMR in cardiac transplant recipients (Gassen et al., 2022). Furthermore, it was clinically confirmed that the ratio of Tfh cells increased in the peripheral blood of transplant recipients with AMR (Liu et al., 2022). These findings indicate that Tfh cells play an important role in the AMR. Moreover, Tfh cells and humoral immunity inhibit the long-term survival of grafts (Montgomery et al., 2011). The mature GC is essential for the function of Tfh cells, and the disruption of GCs has been shown to prolong long-term survival in the murine chronic cardiac transplant rejection model (Chhabra et al., 2018). Another study showed that blocking the generation of Tfh cells through knockout of mammalian target of rapamycin (mTOR) improved the long-term survival of cardiac grafts (Xie et al., 2020). These findings suggest that Tfh cells may be an effective target for the inhibition of AMR and the prolongation of the long-term survival of cardiac grafts.

B cell lymphoma 6 (BCL6) is the characteristic transcription factor of Tfh cells, induces the expression of Tfh cell-related genes during the differentiation of CD4^+^ T cells and inhibits T(H) lineage cell differentiation (Nurieva et al., 2009; Crotty, 2011). BCL6 knockout in T cells impairs the development of Tfh cells and GC responses, suggesting that BCL6 is a significant target for the intervention of Tfh cells (Zhang et al., 2021; Song et al., 2022). T cells enter the follicular zone dependent on the chemokine receptor CXCR5(22). T cells deficient in BCL6 cannot express CXCR5 but can impinge on the calcium signaling of Tfh cells, thereby controlling the synaptic and adhesive interactions between Tfh and B cells. In this way, BCL6 impacts the functions of Tfh cells (Morita et al., 2011; Liu et al., 2021). BCL6 knockout also influence the generation of other effector T-cell subsets (Okada et al., 2012; Choi and Crotty, 2021), however, the effect of BCL6 knockout on cardiac grafts has not yet been reported. Compared with gene editing, the intervention method with small molecule inhibitors is easy for clinical translational applications. The BCL6 inhibitor BI-3802 can accelerate the ubiquitination and degradation of BCL6 (Slabicki et al., 2020). In contrast, the inhibitor FX1, which was shown to be a promising target in lymphocytes, can disrupt the formation of the BCL6 repression complex and efficiently suppress the transcriptional function of BCL6 (Cardenas et al., 2016). FX1 effectively inhibits the generation of Tfh cells in both autoimmune disease and tumor models, indicating its effective interventional activity in Tfh cells (Venkatadri et al., 2022).

In the current study, the murine heterotopic cardiac transplant model was established, and the preparation of Tfh cells were measured under the conditions of acute and chronic cardiac transplant rejection. In the model mice, Tfh cells appeared at 7 days after transplantation, and their numbers gradually increased in the presence of antigens. FX1 prolonged graft survival in chronic rejection but was ineffective in acute rejection. Compared with the control mice, the mice administered FX1 exhibited a significantly decreased preparation of Tfh cells and decreased levels of mature B cells and antibodies. In conclusion, FX1 attenuated humoral immune responses and suppressed chronic rejection, suggesting that BCL6 is a potential therapeutic target of the treatment for chronic cardiac transplant rejection.

## 2 Methods

### 2.1 Animals

BALB/c (B/c, H-2d) and C57BL/6 (B6, H-2b) mice were obtained from Hebei Medical University (Shijiazhuang, Hebei, China). Eight-week-old male mice weighing 20–30 g were selected for this study. All animals were kept and feeded in special pathogen-free (SPF) laboratories at Hebei Medical University Animal Center. The animal experiment of this article was approved by the Animal Ethics Committee of Hebei Medical University.

### 2.2 Hematoxylin and eosin, elastica van Gieson and Masson staining

Mouse tissues were harvested, fixed in 4% paraformaldehyde (PFA) overnight, embedded in paraffin, and sectioned. We performed hematoxylin and eosin (H&E) staining according to the methods outlined in previous studies (Tian et al., 2021). Cellular rejection grading guidelines from the 2004 International Society for Heart and Lung Transplantation were used to grade the rejection response of the cardiac allografts (Mehra et al., 2010). Chronic rejection tissue sections were stained with elastica van Gieson stain. Tissue fibrosis was detected using Masson staining. Deparaffinized and rehydrated slides were incubated with a Masson staining solution for 5 min, followed by 6.5 min of phosphomolybdic acid-aniline blue staining. Image-Pro Plus 6.0 was used to calculate the area of blue-stained collagen fibers within the total field of view.

### 2.3 Heart transplantation model in mice

Murine heart transplants were performed according to the previously described method (Wu et al., 2017). Briefly, donor hearts were harvested from B/c mice and transplant into recipient B6 mice. In detail, B/c`s aortas and pulmonary arteries were anastomosed with the abdominal aorta and inferior vena cava of recipients. Heart allografts were monitored daily by palpation, and graft arrest was considered rejection. Mice were exposed to either vehicle (PBS) or FX1 12 h after surgery and treated for 6 days with 25 mg/kg FX1 every 3 days. The control group received only DMSO injections. Single-dose CTLA4-Ig treatment (250 µg on day 1) of the recipients resulted in chronic rejection.

### 2.4 Flow cytometry assay

The spleens of the recipient mice were collected and stained for flow cytometry (FCM) assay. The spleens were mechanically ground into single-cell suspensions by slide mechanical grinding before staining. The eBioscience Fixable Viability solution was used to exclude dead cells. To stain T and B cells, additional antibodies against CD45, CD3, CD8, CD4, PD1, CXCR5, FOXP3, CD44, CD62L, Ki67, BCL6, B138, CD19, GL7, CD95, PNA, IgM, and IgG1 were used (from eBioscience and BioLegend). Following the manufacturer’s instructions, we used the FOXP3 intracellular buffer (from eBioscience) for intracellular staining. BioLegend provided all the antibodies used in this study.

### 2.5 Donor-specific antibody (DSA) measurement

B/c or B6 mice were uesed as probes for measuring donor-specific antibodies in the sera of the recipients. After washed two times with PBS, 5 × 10^5^ spleen cells were added into 96-well plates. Next, cells were incubated with serum from mice model for half an hour (1:50 dilution). We then stained the cells with Fixable Viability Dye (from eBioscience), anti-B220 (from BioLegend). Meanwhile, anti-CD3 (from BioLegend) were added with Fc-block (from BioLegend). We washed the cells twice and incubated them with anti-IgM as well as anti-IgG antibodies for 30 min. The donors’ reactive antibodies against MHC class II were assessed by measuring anti-IgG levels or the anti-IgM signal on total B220^+^ cells. The mean fluorescence intensity (MFI) of the anti-IgG or anti-IgM signals on the total CD3^+^ cells was assessed by FCM.

### 2.6 Statistical analysis

The Kolmogorov–Smirnoff test was used to determine whether the data were normally distributed. We analyzed differences between groups using the two-tailed unpaired Student’s t-test for normally distributed data. We used Mann-Whitney U-test for non-normally distributed data, and we used Student’s t-test with Welch’s correction when variances were different. We analyzed differences between groups using one-way ANOVA for more than two groups, followed by Tukey corrections. This experiment was repeated 3 times with similar results and a representative experiment is shown. In all tests, 0.05 was considered significant, and the data are presented as means ± SD (standard deviations).

## 3 Results

### 3.1 The expansion of Tfh cells during acute cardiac transplant rejection in mice

**FIGURE 1:**
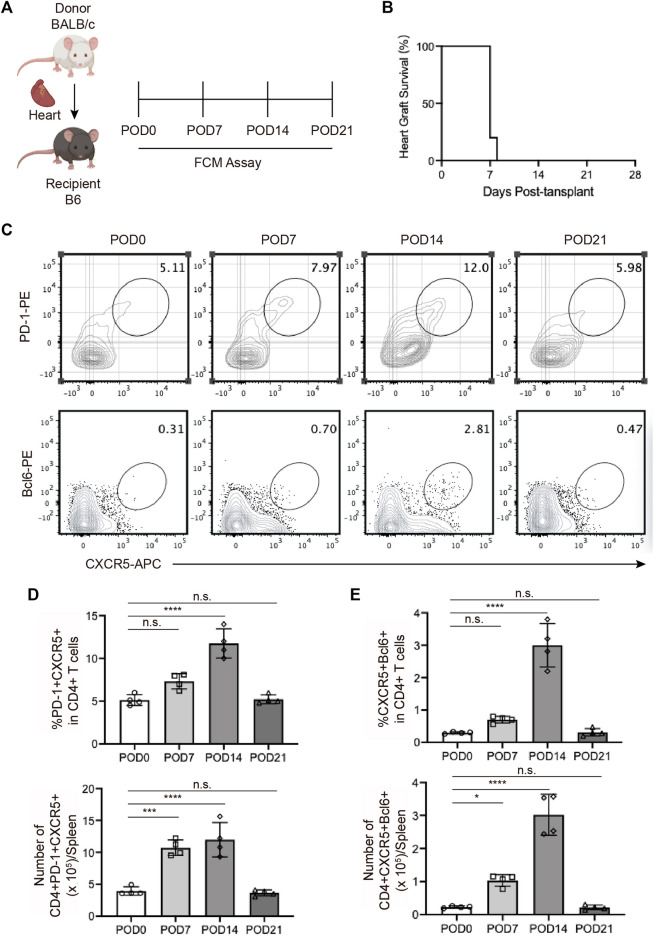
The expansion of T follicular helper cells during acute cardiac transplant rejection in mice **(A)**. Illustration of the experimental design in **(B–E)**. The murine intra-abdominal heterotopic cardiac transplantation model (BALB/c to B6 mice) was established, and the spleens of recipient mice were collected for flow detection at 0, 7, 14, and 21 days post-transplantation **(B)**. The survival status of the grafts was observed every day through palpation. The survival time is illustrated in the survival curves **(C)**. FCM analysis of the proportions of PD1+CXCR5+ Tfh and BCL6+CXCR5+ Tfh in the spleens of recipient mice at 0, 7, 14, and 21 days post-transplantation **(D)**. The bar charts (*n* = 4) show the ratios and numbers of PD1+CXCR5+ Tfh cells in the spleens at different time points, as described in 1a **(E)**. The bar charts (*n* = 4) show the ratios and numbers of BCL6+CXCR5+ Tfh cells in the spleens at different time points, as described in 1a.Error bars represent SD. ns, not significant, *p < 0.05, **p < 0.01, ***p < 0.001, ****p < 0.0001.

The changing trend of Tfh cell ratio over time in animal models has not yet been investigated. In the current study, we constructed the murine heterotopic cardiac transplant model following the reported methods to simulate acute rejection (Figure 1A). The cardiac grafts ceased beating 1 week after transplantation (Figure 1B), consistent with previously reported results. We detected the ratios and numbers of Tfh cells in mouse spleens 0, 7, 14, and 21 days after transplantation *via* FCM and found that the ratios of PD-1^+^CXCR5^+^ Tfh and BCL6^+^CXCR5^+^ Tfh cells both began to increase on the 7th postoperative day, peaked on the 14th day, and rapidly decreased on the 21st day (Figures 1C, D, E). The differentiation of Tfh cells was not completed when graft dysfunction occurred.

### 3.2 The expansion of Tfhs during chronic cardiac transplant rejection in mice

**FIGURE 2:**
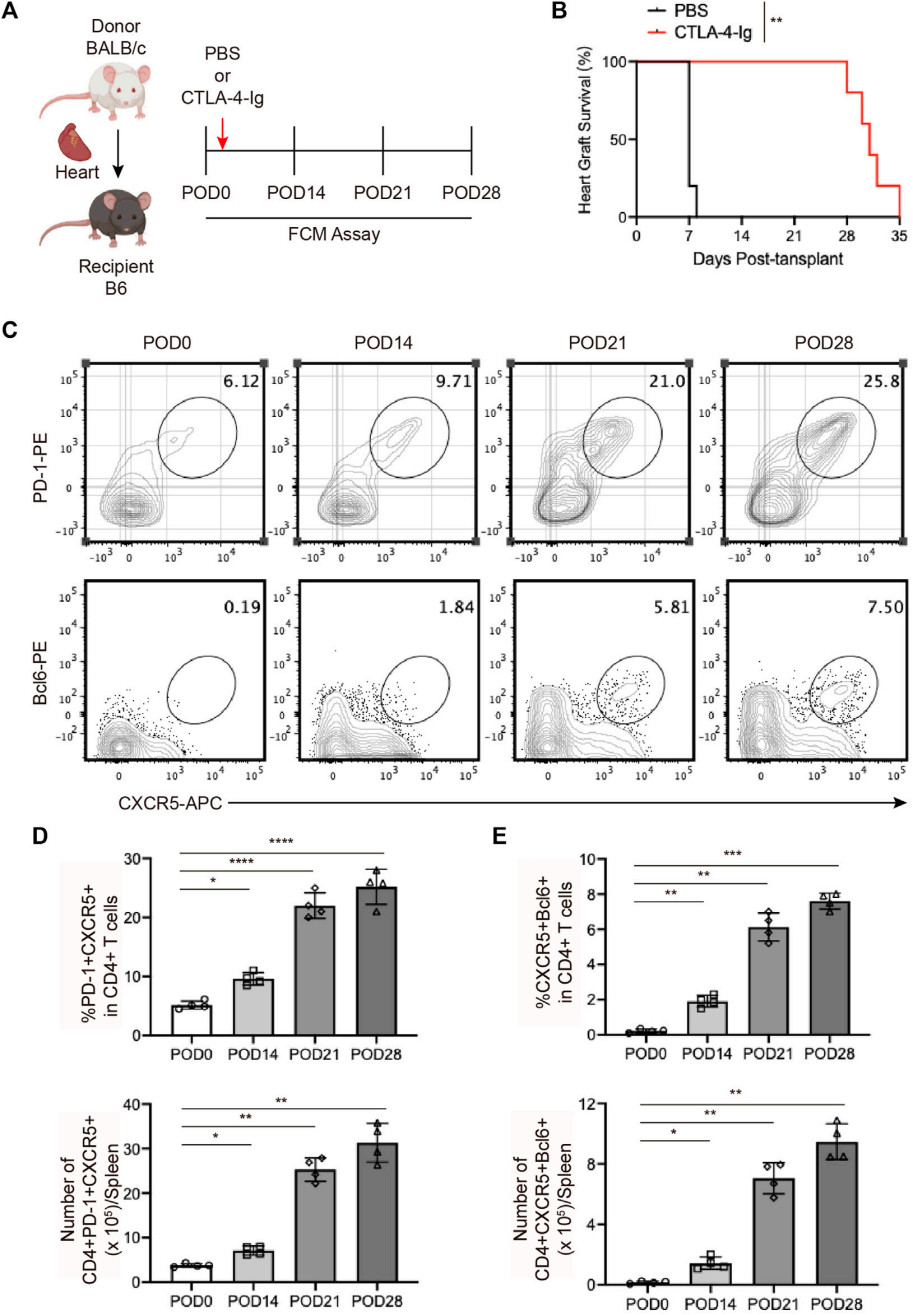
The expansion of T follicular helper cells during chronic cardiac transplant rejection in mice **(A)**. Illustration of the experimental design in **(B–E)**. The murine chronic cardiac transplant model was established. The mice were injected with 250 ug of CTLA-4-Ig or the equivalent amount of PBS intraperitoneally on the day after surgery, and their spleens were detected *via* FCM at 0, 7, 14, and 21 days post-transplantation. AuthorAnonymous, **(B)**. The survival status of the grafts was observed every day through palpation. The survival time is illustrated in the survival curves (*n* = 6) **(C)**. FCM analysis of the proportions of PD1+CXCR5+ Tfh and BCL6+CXCR5+ Tfh cells in the spleens of recipient mice at 0, 7, 14, and 21 days post-transplantation **(D)**. The bar charts (*n* = 4) show the ratios and numbers of PD1+CXCR5+ Tfh cells in the spleens at different time points, as described in 2a **(E)**. The bar charts (*n* = 4) show the ratios and numbers of BCL6+CXCR5+ Tfh cells in the spleens at different time points, as described in 2a.Error bars represent SD. ns, not significant, *p < 0.05, **p < 0.01, ***p < 0.001, ****p < 0.0001.

To explore the role of Tfhs in chronic cardiac transplant rejection, we established the murine heart transplantation with the BALB/c mice as the donor and B6 mice as the recipient, and treated with CTLA-4-Ig as previous reported (Figure 2A) (Borges et al., 2021). As shown in Figure 2B, we found the cardiac grafts could survive for over 4 weeks with the CTLA-4-Ig treatment (Figure 2B). On days 14, 27, 28 post-transplants, the splenocytes cells from recipient were collected for FCM analysis. We found that the proportion of PD-1^+^CXCR5^+^ Tfh and BCL6^+^CXCR5^+^ Tfh cells were increased over time and peaked on the 28th postoperative day (Figures 2C, D, E). The continuously elevated proportion of Tfh cells might have been associated with the stimulation of antigens.

### 3.3 Small-molecule BCL inhibitor FX1 does not protect acute cardiac transplant rejection in mice, but it inhibits Tfhs expansion

**FIGURE 3:**
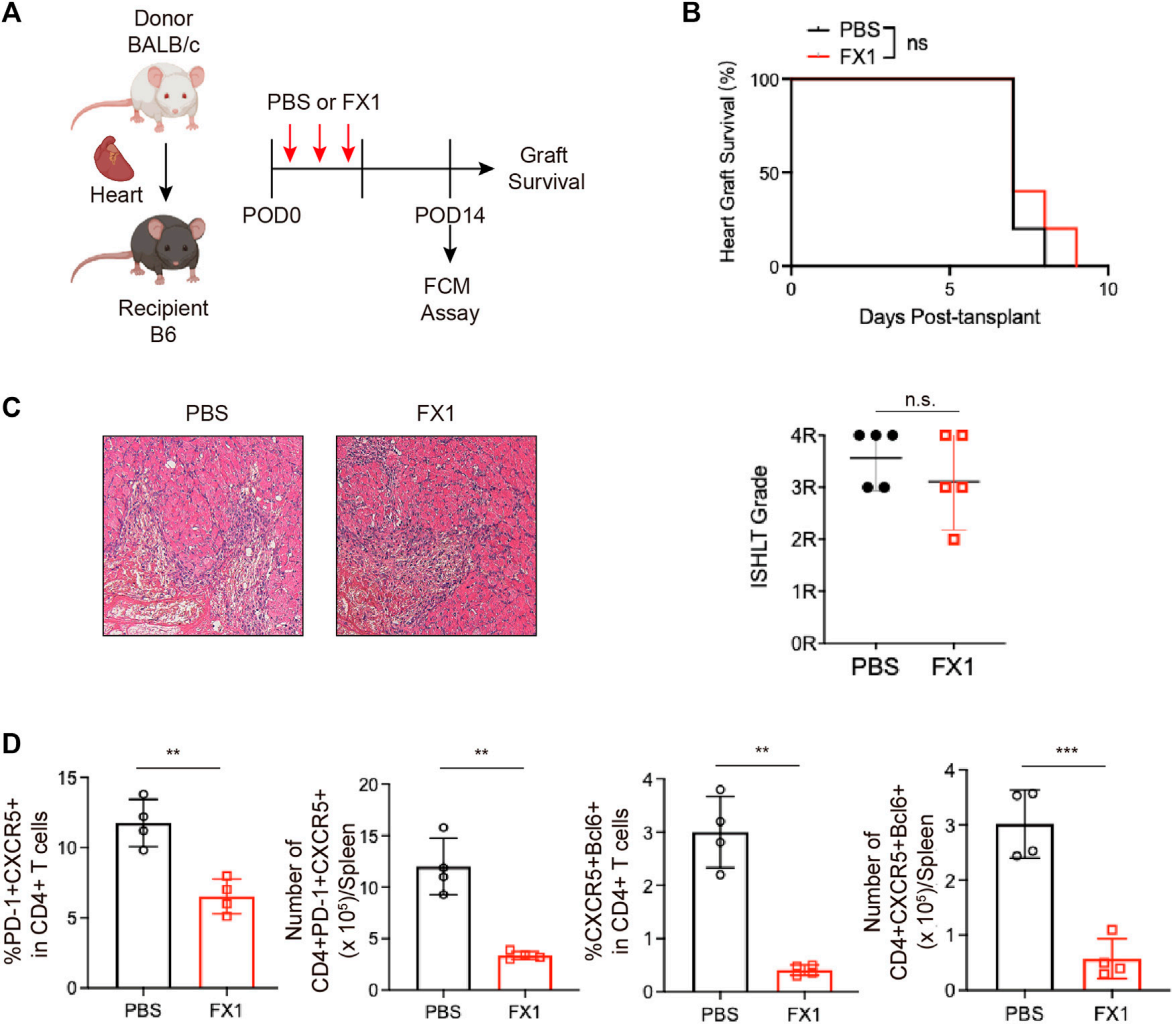
Small-molecule BCL inhibitor FX1 does not protect acute cardiac transplant rejection in mice, but it inhibits T follicular helper cell expansion **(A)**. Illustration of the experimental design in **(B–E)**. The murine acute cardiac transplant model was established, and the mice were administered CTLA-4-Ig or the equivalent amount of PBS for 3 consecutive days. The survival time of the cardiac grafts was observed, and the spleens were collected for FCM analysis on the 14th postoperative day **(B)**. The survival status of the grafts was observed every day through palpation, and the survival time is illustrated in the survival curves (*n* = 6) **(C)**. Graft specimens were obtained on the 6th postoperative day, and they were stained with H&E to visualize pathological changes in the grafts. International Society for Heart and Lung Transplantation grading was performed on tissue sections of each group (*n* = 6) **(D)**. The bar charts (*n* = 4) show the ratios and numbers of PD1+CXCR5+ Tfh and BCL6+CXCR5+ Tfh cells in the spleens on the 6th day post-transplantation. Error bars represent SD. ns, not significant, *p < 0.05, **p < 0.01, ***p < 0.001, ****p < 0.0001.

The small molecule inhibitor FX1 has been shown in multiple studies to be a safe and effective BCL6 inhibitor. Based on previous research, the mice with acute rejection were administered three injections of FX1 or control (Figure 3A). Their graft conditions were observed, and Tfh cell levels were detected. We discovered that FX1 did not prolong the survival of grafts (Figure 3B). The grafts were removed and fixed for H&E staining on the sixth postoperative day. Staining revealed no obvious differences in the pathological changes between the graft sections of the two groups, which had approximately identical pathological scores (Figure 3C). Given that the spleen Tfh cell level peaked on the 14th postoperative day, we measured spleen lymphocyte levels during the 14 postoperative days with FCM and found that FX1 administration decreased the numbers and ratios of PD-1^+^CXCR5^+^ Tfh and BCL6^+^CXCR5^+^ Tfh cells (Figure 3D). These findings suggest that FX1 did not protect the cardiac grafts from acute rejection in mice but that it inhibited the expansion of Tfh cells.

### 3.4 FX1 protects chronic cardiac transplant rejection in mice

**FIGURE 4:**
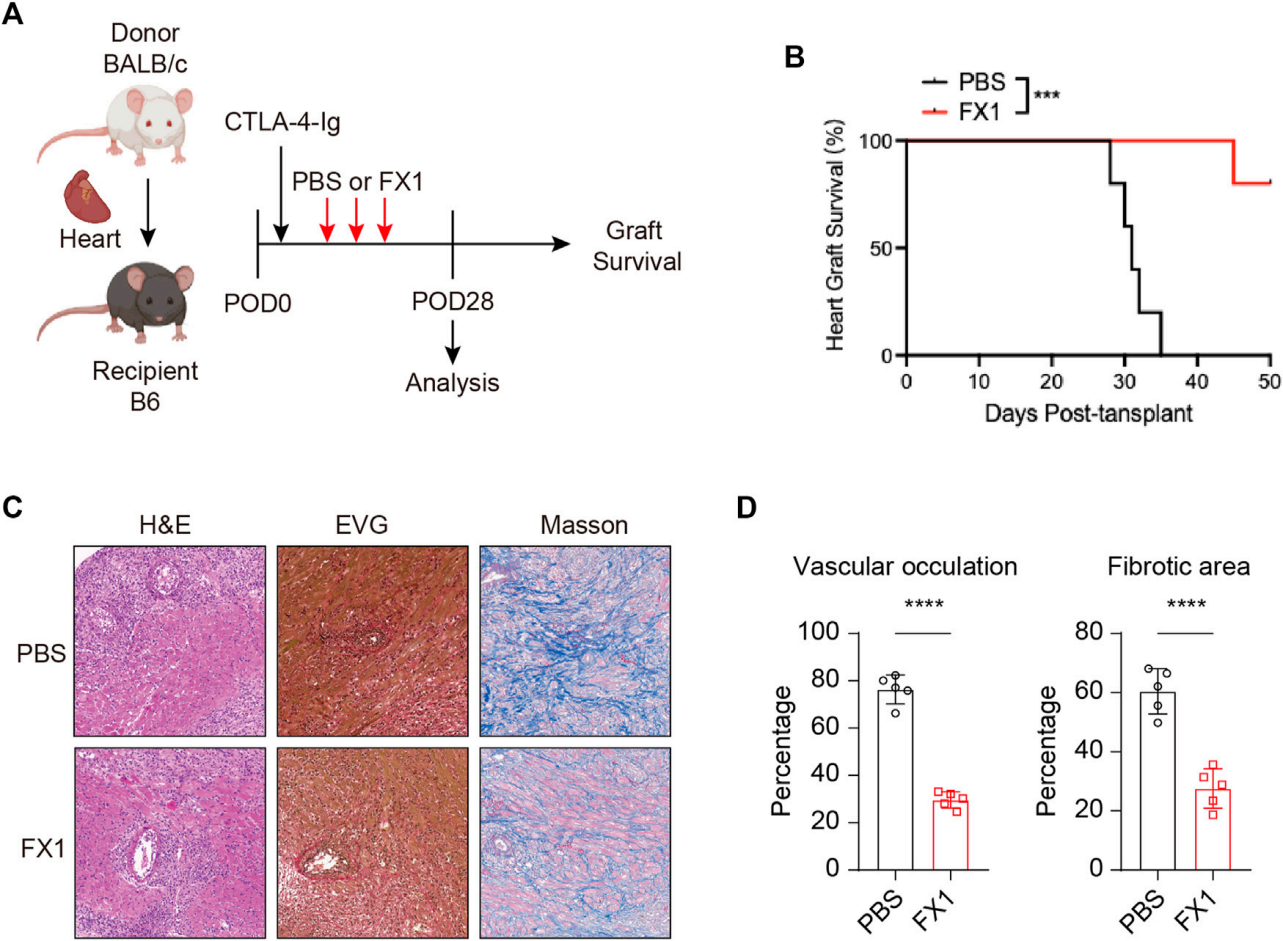
FX1 protects chronic cardiac transplant rejection in mice **(A)**. Illustration of the experimental design in **(B–E)**. The murine chronic cardiac transplant model was established, and the mice were treated with FX1 or the equivalent amount of PBS three times. The survival time of the cardiac grafts was observed, and the spleens were collected for FCM analysis on the 28th postoperative day **(B)**. The survival status of the grafts was observed every day through palpation, and the survival time is illustrated in the survival curves (*n* = 6) **(C)**. Graft specimens were obtained on the 28th postoperative day, and vascular occlusion and fibrosis in the cardiac grafts was assessed by H&E, elastica van Gieson, and Masson staining **(D)**. The bar charts shows the vascular occlusion and fibrosis of the cardiac grafts (*n* = 6). Error bars represent SD. ns, not significant, *p < 0.05, **p < 0.01, ***p < 0.001, ****p < 0.0001.

As the characteristics of Tfh cells in recipient mice after heart transplantation, we explored the effect of FX1 on chronic cardiac transplant rejection in mice. As abovementioned method, we construct heart transplant model with chronic rejection and treat with FX1 at 25 mg/kg FX1 every 3 days (Figure 4A). We found that FX1 administration significantly prolonged the survival of cardiac grafts and suppressed chronic rejection (Figure 4B). The H&E staining of cardiac grafts revealed that the infiltration of lymphocyte and damage of cardiomyocyte in cardiac graft were obviously reduced with the FX1 treatment (Figure 4C). The EVG and Masson staining of cardiac grafts showed that vascular occultation and myocardial interstitial fibrosis of cardiac graft were significantly inhibited in the FX1 treated mice (Figures 4C, D). These findings indicate that FX1 protects cardiac grafts from chronic rejection in mice.

### 3.5 FX1 inhibits Tfhs cell expansion during chronic cardiac transplant rejection in mice

**FIGURE 5:**
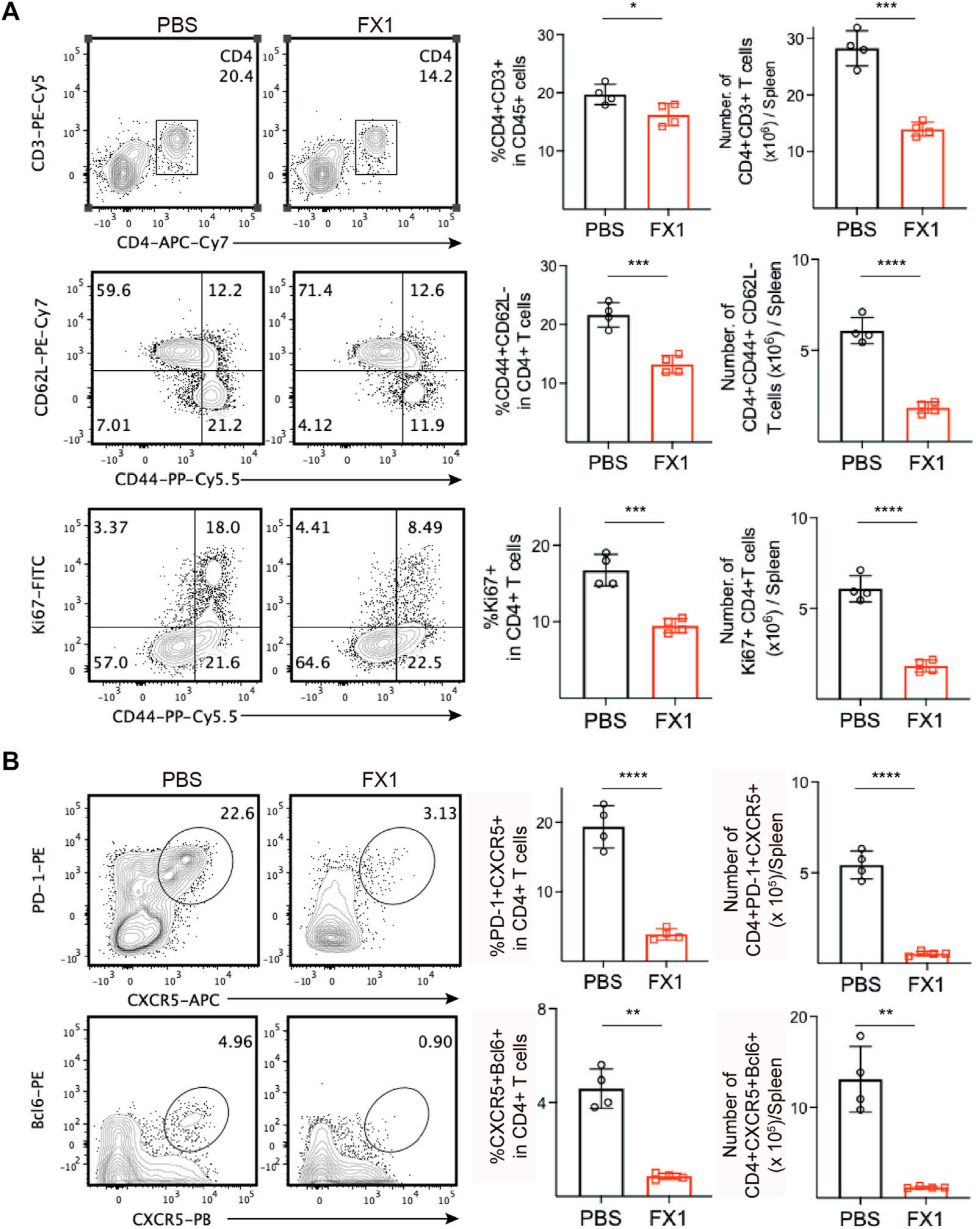
FX1 inhibits T follicular helper cell expansion during chronic cardiac transplant rejection in mice **(A)**. The spleens were obtained from the recipient mice on postoperative day 28 and were ground into single-cell suspensions for FCM detection. The contour plots demonstrate the ratios of characteristic CD3^+^CD4^+^ T cells, CD44^+^CD62L-T cells, and CD44+Ki67 + T cells in both groups, while the bar charts (*n* = 4) illustrate the ratios between the two groups **(B)**. The contour plots demonstrate the ratios of characteristic PD1+CXCR5+ Tfh and BCL6+CXCR5+ Tfh in both groups, while the bar charts (*n* = 4) illustrate the ratios of Tfh cells between the two groups. Error bars represent SD. ns, not significant, *p < 0.05, **p < 0.01, ***p < 0.001, ****p < 0.0001.

We identified mouse spleens *via* FCM to further investigate the effect of FX1 on chronic rejection. Initially, we found that the FX1-treated mice had a decreased ratio and number of splenic CD4^+^ T cells. Subsequently, the conditions of the CD4^+^ T cells were monitored, and we discovered that the numbers and ratios of effector memory T (TEM) cells and proliferating Ki67^+^CD44^+^CD4 T cells decreased significantly in the FX1-treated mice (Figure 5A), indicating that FX1 substantially attenuated the responses of CD4^+^ T cells in mice with chronic rejection. In addition, the numbers and ratios of PD-1^+^CXCR5^+^ Tfh and BCL6^+^CXCR5^+^ Tfh cells were decreased, in line with our previous findings (Figure 5B). These findings demonstrate that FX1 inhibits Tfh cell expansion during chronic cardiac transplant rejection in mice.

### 3.6 FX1 inhibits T follicular helper cell expansion during chronic cardiac transplant rejection in mice

**FIGURE 6:**
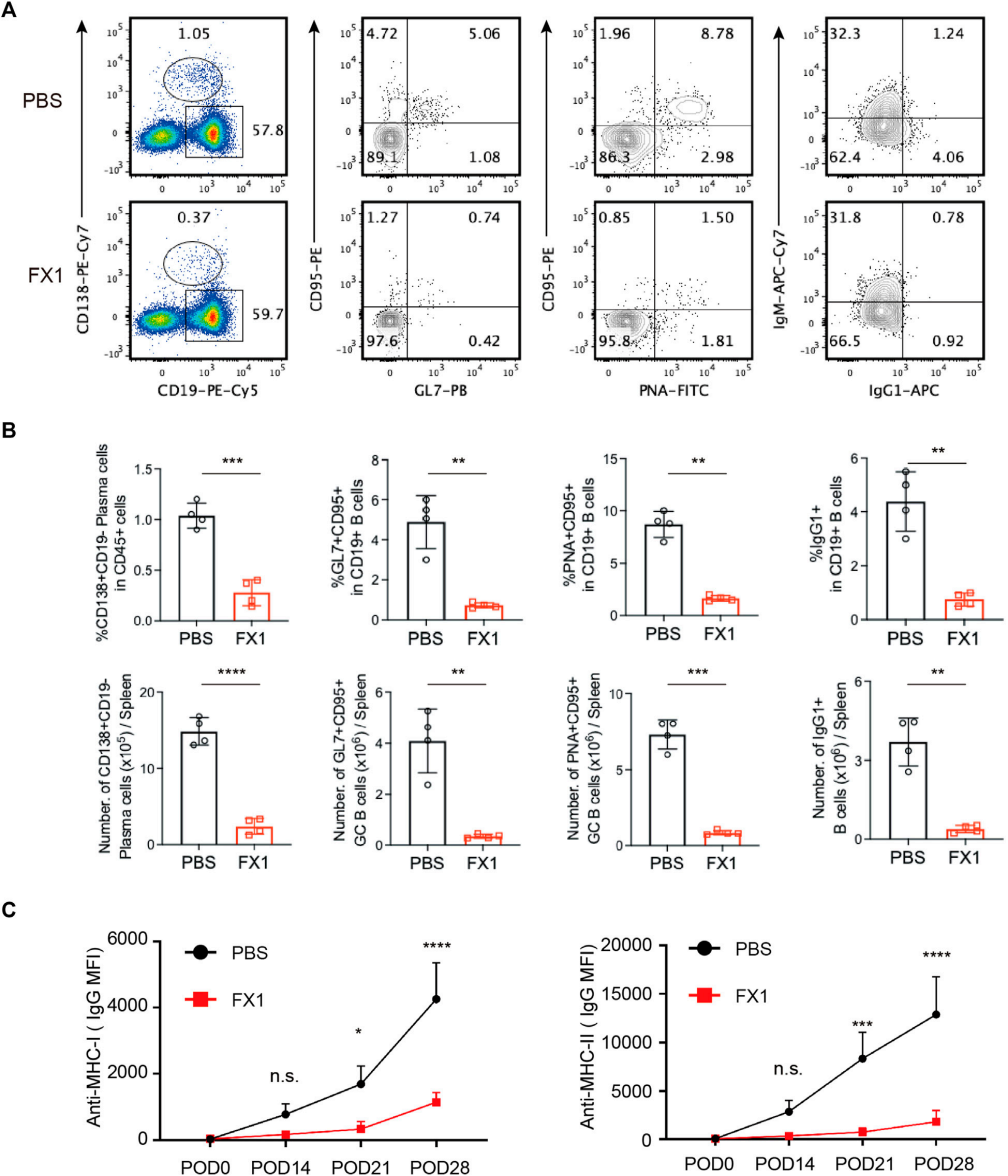
FX1 inhibits humoral response during chronic cardiac transplant rejection in mice **(A)**. Spleens were obtained from the recipient mice on postoperative day 28, ground into single-cell suspensions for FCM detection, and stained for the ratios of CD19^−^CD138+ plasma cells, GL7+CD95+B cells, PNA+CD95+B cells, and IgG1+CD19+B cells **(B)**. The bar charts (*n* = 4) illustrate the ratios and number of CD19^−^CD138+ plasma cells, GL7+CD95^+^ GC B cells, PNA+CD95^+^ GC B cells, and IgG1+CD19+B cells in both groups **(C)**. The sera of the mice in both groups were collected 0, 14, 21, and 28 days postoperatively to measure the concentrations of anti-MHC-I and anti-MHC-II antibodies.

To investigate whether FX1 affects humoral immunity in chronic rejection, B lymphocytes were examined. The ratio of plasma cells was measured first as these cells play the dominant role in performing humoral immune functions. Following FX1 administration, the ratio of CD19^+^CD138^+^ plasma cells significantly decreased, and the number of plasma cells also decreased (Figures 6A, B). As Tfh cells interact with and affect the functions of B cells in GCs, the changes in GC B cells were tracked. It was discovered that among the FX1-treated mice, the ratios and numbers of GL7^+^CD95^+^ and PNA^+^CD95^+^ B cells decreased, and the number of B cells in GCs also decreased (Figures 6A, B). Furthermore, the ratio of IgG1^+^ memory B cells substantially decreased, and the number of memory B cells also decreased following FX1 administration (Figures 6A, B). Finally, the ratios of alloantibodies 0, 14, 21, and 28 days post-transplantation were assessed. The FX1-treated mice exhibited decreased ratios of anti-MHC-I and anti-MHC-II antibodies (Figure 6C) in addition to compromised humoral immunity. These findings suggest that FX1 inhibits the humoral response during chronic cardiac transplant rejection in mice.

## 4 Discussion

The current study explored the effect of the BCL6 inhibitor FX1 on acute and chronic cardiac transplant rejection in mice. In acute rejection, Tfh cells appeared late. Although FX1 inhibited the generation of Tfh cells, it did not affect the outcomes of the grafts. In chronic rejection, the survival of the grafts was substantially prolonged, the proportion of Tfh cells was decreased and humoral immunity was impaired with the FX1 treatment. Our study demonstrated that the BCL6 is a therapeutic target for chronic cardiac graft rejection and validated the effectiveness of FX1 treatment after transplantation.

Graft dysfunction had already occurred when Tfh cells appeared during acute rejection, suggesting the non-significant effect of Tfh cells on acute rejection (Dudreuilh et al., 2020). This may explain why FX1 did not protect cardiac grafts from acute rejection. In a previous study, among the murine experimental autoimmune encephalomyelitis (EAE) model once the mice were immunized with the myelin oligodendrocyte glycoprotein (MOG) peptide, the proportion of Tfh cells on the 7th day post-immunization was far lower than on the 14th day, and it decreased again on the 21st day (Zotos et al., 2010), which was in line with our findings. In contrast, in another study, due to the long duration of EAE in the murine model and humoral immunity was involved in the inflammatory response of the disease. Therefore, the treatment of targeting Tfh cells protected the EAE model (Varrin-Doyer et al., 2016). In the current study, the number of Tfh cells continued to increase over time in chronic rejection, which might be due to the persistence of antigens. Similarly, in the lymphocytic choriomeningitis virus model in which antigens are continuously present, the proportion of Tfh cells also increases over time. Moreover, as viral antigens also persist in the lymphocytic choriomeningitis virus model and humoral immunity is the most crucial part of anti-viral immunity, Tfh cells has been an important target in anti-viral immunity (Leong et al., 2016). In a recent clinical study on renal transplantation, the renal biopsies of patients who experienced renal transplant rejection revealed the co-localization of Tfh and B cells, indicating that T cells could induce B cells to produce alloantigen-specific antibodies, which might lead to clinical graft dysfunction (de Graav et al., 2015). In blood samples from cardiac transplant recipients, the long-term Tfh cell proportion was increased, strengthening the responses of B cells. In addition, the Tfh-related cytokines CXCL13 and IL-21 were shown to accelerate the immunopathogenesis of chronic immune damage (Wang et al., 2020). Blazar et al. established the murine hematopoietic stem cell transplantation model and revealed that Tfh cells were necessary for the development of chronic graft-versus-host disease (cGVHD) in mice (Flynn et al., 2014). Therefore, Tfh cells serve as an important target for the regulation of long-term allograft survival.

The BCL6 inhibitor FX1 has been extensively used. Evidence from a study on B-cell lymphoma indicated that MLL-rearranged acute lymphoblastic leukemia could be treated with FX1, with its safety and efficacy validated at the animal level (Hurtz et al., 2019). Moreover, FX1 alleviated erythromycin resistance in acute lymphoblastic leukemia, serving as an adjunctive medication in anti-tumor therapy (Tsuzuki et al., 2022). In the murine sepsis model, FX1 exhibited extraordinary anti-inflammatory capacity (Zhang et al., 2019). These findings suggest that the FX1 is sufficient to suppress BCL6 *in vivo*. In addition to inhibiting BCL6 expression and decreasing peripheral Tfh content in primates (Cai et al., 2020), FX1 demonstrated favorable therapeutic effects in human-derived cells (Kawabata et al., 2021). Because FX1 was shown to protect cardiac grafts under chronic rejection in mice in our study, we believe that FX1 represents a novel therapeutic target for rejection, which has a promising clinical translational potential. We found that CTLA-4-Ig combined with FX1 increases its efficacy, which we expect will boost its therapeutic efficacy in renal transplant recipients, the therapeutic scope of CTLA-4-Ig may even be expanded to include cardiac transplant recipients. In conclusion, FX1 serves as a promising therapeutic target in the treatment of AMR.

In conclusion, we highlighted the significance of targeting BCL6 in the protection of long-term graft survival. In addition, we discovered that FX1 attenuates humoral immunity and prolongs the long-term survival of cardiac grafts, shedding new light on the treatment of AMR.

## Data Availability

The raw data supporting the conclusion of this article will be made available by the authors, without undue reservation.
