# Supplementary material for: Small-molecule BCL6 inhibitor protects chronic cardiac transplant rejection and inhibits T follicular helper cell expansion and humoral response
Source: Front Pharmacol. 2023 Mar 17;14:1140703. doi: 10.3389/fphar.2023.1140703 (PMC10063191; doi:10.3389/fphar.2023.1140703)
Supplement: Supplementary file 1 [file Table1.DOCX]

**Small-Molecule BCL6 Inhibitor Protects Chronic Cardiac Transplant Rejection and Inhibits T Follicular Helper Cell Expansion and Humoral Response**

Yuxuan Xia^1^, Sheng Jin^1^, Yuming Wu^1,2^*

^1^Department of Physiology, Hebei Medical University, Hebei, 050017, China

^2^Hebei Collaborative Innovation Center for Cardio-Cerebrovascular Disease, 050017, Hebei, China

Supplementary Material

# Supplementary Figure and Figure Legend


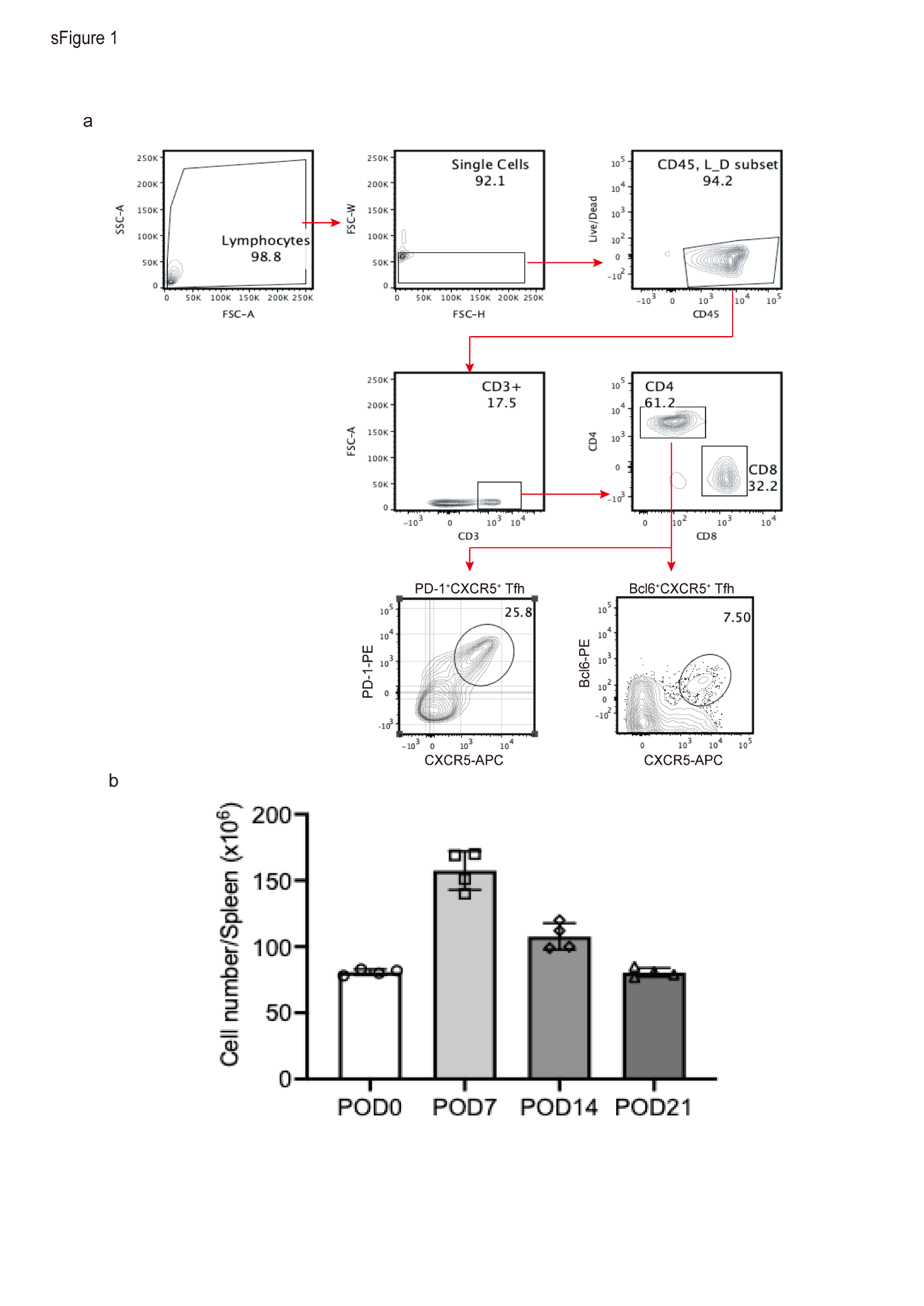


**sFigure1. Gating strategy for splenic Tfh and the number of cells in the spleen****.**

a. Gating strategy for PD1^+^CXCR5^+^ Tfh and BCL6^+^CXCR5^+^ Tfh cells in the recipients’ spleens.

b. Statistical graphs of cell numbers in mouse spleens at different time points during acute cardiac transplant rejection.
